# A cecropin-like antimicrobial peptide with anti-inflammatory activity from the black fly salivary glands

**DOI:** 10.1186/s13071-015-1176-8

**Published:** 2015-10-24

**Authors:** Jing Wu, Lixian Mu, Li Zhuang, Yi Han, Tong Liu, Jun Li, Yuan Yang, Hailong Yang, Lin Wei

**Affiliations:** School of Basic Medical Sciences, Kunming Medical University, 1168 West Chunrong Road, Yuhua Avenue, Chenggong District, Kunming, 650500 Yunnan China; Jiangsu Key Laboratory of Infection and Immunity, Institute of Biology and Medical Sciences, Soochow University, Suzhou, Jiangsu China; The Second Department of Internal Medicine, the Third Affiliated Hospital of Kunming Medical University, Kunming, Yunnan China

**Keywords:** Cecropin, Antimicrobial peptide, Anti-inflammation, Black fly, *Simulium bannaense*

## Abstract

**Background:**

Several antimicrobial peptides (AMPs) belonging to the cecropin family have been identified from the salivary glands of different black fly species, however, the immunological functions for these molecules were poorly understood.

**Methods:**

A novel cecropin-like antimicrobial peptide (*Siba*Cec) was purified using reverse phase high-performance liquid chromatography (RP-HPLC) from the salivary glands of the black fly *Simulium bannaense*. The amino acid sequence of *Siba*Cec was determined by a combination method of automated Edman degradation and cDNA sequencing. The morphologic changes of Gram-negative bacteria *Escherichia coli* treated with *Siba*Cec were assessed by scanning electron microscopy (SEM). Quantitative PCR (qPCR) was performed to analyze the mRNA expression of the inducible NO synthase (iNOS) and pro-inflammatory cytokines. Nitric oxide (NO) generation was examined using a Griess assay and the secretion of pro-inflammatory cytokines was determined by an enzyme-linked immunosorbent assay (ELISA). The activation of extracellular signal-regulated kinase (ERK), p38, and the nuclear translocation of nuclear factor-kappaB (NF-κB) were assessed by Western blotting analysis. Circular dichroism (CD) spectroscopy was performed to evaluate the secondary structure of *Siba*Cec in solvent environment. Interaction of *Siba*Cec with lipopolysaccharide (LPS) was studied using fluorescein isothiocyanate (FITC)- conjugated LPS aggregates. Neutralization of LPS by *Siba*Cec was assayed with the chromogenic limulus amebocyte lysate (LAL) test. qPCR was also used to analyze the expression of *Siba*Cec mRNA in the salivary glands of insects after oral infection with the bacteria *E.coli.*

**Results:**

*Siba*Cec possessed potent antimicrobial activity against Gram-negative bacteria, and showed low cytotoxicity toward mammalian cells. SEM analysis indicated that *Siba*Cec killed bacteria through the disruption of cell membrane integrity. Furthermore, *Siba*Cec significantly inhibited lipopolysaccharide (LPS)-induced production of NO and pro-inflammatory cytokines such as tumor necrosis factor-α (TNF-α), interferon-1β (IL-1β) and interferon-6 (IL-6) by blocking the activation of MAPKs and NF-κB signaling pathways. It mainly adopted an α-helix conformation in membrane-mimetic environments. *Siba*Cec could interact and neutralize LPS. Infection of black flies with bacteria caused an upregulation of the expression of *Siba*Cec.

**Conclusions:**

These results demonstrated that in addition to the bactericidal capacity, *Siba*Cec can function as immune regulator, inhibiting host secretion of inflammatory factors.

**Electronic supplementary material:**

The online version of this article (doi:10.1186/s13071-015-1176-8) contains supplementary material, which is available to authorized users.

## Background

Black flies (Diptera: Simuliidae) are annoying biting pests of humans and animals. They serve as obligate vectors for serious diseases such as human onchocerciasis (river blindness) and numerous arboviruses of livestock [1]. A number of salivary peptides/proteins have been characterized from hematophagous arthropods such as horseflies [2, 3], mosquitoes [4] and ticks [5]. There was relatively limited information available on pharmacologically active compounds in black fly salivary glands, until when the salivary transcriptomes were published for three black fly species (*S. vittatum*, *S. nigrimanum* and *S. guianense*) [6–8]. In these studies, some transcripts related to immunity, including six AMPs of cecropin family, have been revealed by sequence similarities with known peptides/proteins from other organisms. However, there have been very few subsequent efforts to establish the functions of these molecules in black flies. In addition, other immune-related molecules including lectins, prophenoloxidase and antimicrobial peptides/proteins have also been identified from black flies [9–11].

AMPs, including cecropins, defensins, and cathelicidins, are a unique and diverse group of effector molecules that play an important role in humoral immunity in all living organisms [12]. Cecropin was the first insect AMP isolated from the bacteria-challenged *Hyalophora cecropia* pupa [13], and then a number of cecropin-like peptides have been identified from different species of insect orders (Diptera, Lepidoptera, Hymenoptera, Coleoptera and Isoptera) [14, 15] and other organisms including mammals [16]. In insects, cecropin-like peptides have been shown to have effects on bacteria [17], fungi [18], parasites [19] and viruses [20].

The immune-stimulatory/modulatory functions and mechanisms of vertebrate AMPs, especially mammalian AMPs, such as defensins (i.e., hBD-2, hBD-3, and hBD-4) and cathelicidins (i.e., LL-37 and PR-39), have been well studied [21–23]. These AMPs are involved in modulating chemokine and cytokine production in immune cells, altering gene expression in host cells, limiting sepsis, improving wound healing and angiogenesis in vitro and in vivo [24, 25]. Some insect AMPs, such as the AMPs from blood-sucking triatomine bug and midges, have been shown to be involved in the immune responses [26–28]. In fact, there are relatively few studies that focus on the anti-inflammatory functions for these AMPs. To date, two AMPs of cecropin family (papiliocin and cecropin A) with anti-inflammatory activity have been characterized from the swallowtail butterfly *Papilio xuthus* [29] and cecropia moth *Hyalophora cecropia* [30]. Additionally, several hybrid peptides that are composed of cecropin A and other AMPs also showed the same activity [31–33].

We report herein the purification and characterization of a novel cecropin-like peptide with both antimicrobial and anti-inflammatory activities from the salivary glands of the hematophagous insect black fly *S. bannaense*.

## Methods

### Salivary gland dissection

Adult *S. bannaense* were collected near streams in Xishuangbanna, Yunnan, China. As our previous report [11], the black fly salivary glands were dissected in ice cold HEPES saline (10 mM HEPES pH 7.2, 150 mM NaCl) using fine entomological needles under a stereomicroscope, and stored in liquid nitrogen until use.

### Ethical approval

The study was approved by the Animal Care and Use Ethics Committee of Kunming Medical University.

### Peptide purification

**Fig. 1:**
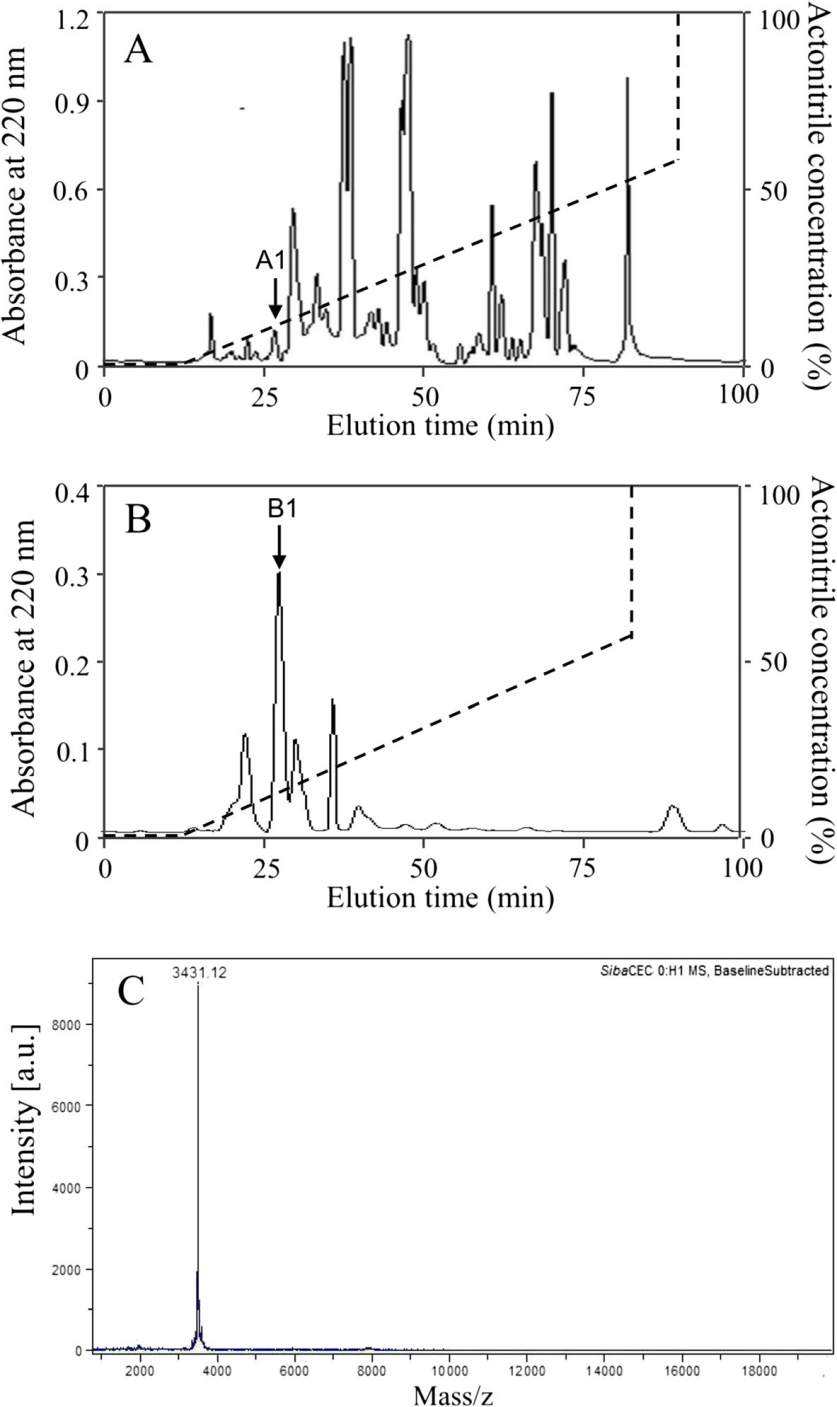
Purification of *Siba*Cec from the salivary gland of *S. bannaense* and MALDI–TOF MS. **a** The filtrate of the salivary gland homogenate of *S. bannaense* was divided by an Inertsil C_4_ RP-HPLC column. **b** The eluted peak of A1 containing antimicrobial activity was further purified by C_18_ RP-HPLC column. The purified *Siba*Cec is indicated by an arrow. **c** MALDI-TOF mass spectrometry analysis of the purified *Siba*Cec

According to the methods in our previous report [11], the eluted peak of A1 (Fig. 1a) containing antimicrobial activity was pooled, lyophilized, and further purified by RP-HPLC on a Wondasil C_18_ column (25 × 0.46 cm). The elution was performed using a linear gradient of 0–60 % acetonitrile containing 0.1 % (v/v) trifluoroacetic acid in 0.1 % (v/v) trifluoroacetic acid/water over 70 min. N-terminal sequence of the purified peptide was done by Edman degradation on an Applied Biosystems pulsed liquid-phase sequencer (model ABI 491).

### MALDI-TOF MS analysis

Matrix-assisted laser desorption/ionization time-of-flight mass spectrometry (MALDI-TOF MS) was used to analyze the purified peptide. AXIMA CFR mass spectrometer (Kratos Analytical) was operated in linear and positive ion mode. Polypeptide mass standard (Kratos Analytical) is used as an external calibration.

### cDNA library construction and cDNA cloning

As our previous report [11], the In-Fusion SMARTer^TM^ Directional cDNA Library Construction Kit (Takara, Japan) was used to prepare cDNAs. A sense degenerate primer (*Siba*Cec-F_1_, according to the sequence determined by Edman degradation) was coupled with a 3’ PCR primer (the adaptor sequence of 3’ In-Fusion SMARTer CDS Primer provided in the kit) to screen the 3’ fragment of cDNA encoding *Siba*Cec. The full length cDNA was finally obtained from primers of *Siba*Cec-R_1_(antisense primer, according to the 3’-coding region of cDNA) and 5’ PCR primer from the library kit. The PCR conditions were: 95 °C for 5 min and 30 cycles of 95 °C (30 s), 56 °C (30 s), 72 °C (60 s) followed by an extension step at 72 °C for 8 min. Primers used in this research are shown in Additional file 1: Table S1.

### Sequence analysis

The theoretical isoelectric point (pI) and molecular weight of *Siba*Cec were analyzed through Bioinformatics Resource Portal (http://www.expasy.org/tools/) [34]. Multi-sequence alignment was performed using ClustalW (http://embnet.vital-it.ch/software/ClustalW.html) [35]. The phylogenetic tree was constructed by the neighbor-joining method in Mega 5 package.

### Antimicrobial assay

The microbicidal activity of *Siba*Cec was tested as described in our previous paper [36]. Standard and clinically isolated drug resistance bacteria were obtained from the First Affiliated Hospital of Kunming Medical University. Minimal inhibitory concentration (MIC) was defined as the lowest concentration at which no visible growth of microorganisms occurred.

### SEM

Bacteria *E. coli* ATCC 25922 were incubated with *Siba*Cec (1 × MIC) diluted in phosphate buffered saline (PBS) at 37 °C for 45 min. Bacteria incubated with PBS was used as negative control. After centrifugation, bacteria pellets were fixed with 2.5 % glutaraldehyde solution for 2 h at 4 °C and then postfixed in 1 % osmium tetroxide for 2 h. Dehydration was carried out with a graded series of alcohols. Bacteria were mounted onto aluminium stubs and sputtered with gold. Images were visualized in a Hitachi S-4800 electron microscope.

### Cytotoxic and hemolytic assay

The cytotoxic activity was carried out as described in our previous work [37]. Briefly, 2 ml of 3 % (w/v) Brewer thioglycollate medium was injected into the peritoneal cavity of C57BL/6 mice. Three days later, peritoneal macrophages were harvested and cultured in 96-well plates (2 × 10^4^ cells/well) in Roswell Park Memorial Institute (RPMI) 1640 medium supplemented with 2 % fetal bovine serum (FBS), 100 U/ml penicillin, and 100 μg/ml streptomycin. *Siba*Cec dissolved in serum-free RPMI 1640 medium were added to wells, and the serum-free RPMI 1640 medium without *Siba*Cec was used as control. After incubation at 37 °C for 24 h, 20 μl of MTT (3-(4,5-dimethylthiazol-2-yl)-2,5-diphenyltetrazolium bromide) solution (5 mg/ml) was added to each well, and the cells were further incubated for 4 h at 37 °C. The cells were dissolved in 200 μl of Me_2_SO, and the absorbance at 570 nm was measured on a microplate reader (Epoch Etock, BioTek, USA).

Hemolytic assay was conducted as previously reported [36]. Serial dilutions of *Siba*Cec were incubated with the washed human erythrocytes at 37 °C for 30 min. After centrifugation, the absorbance of supernatant was measured at 540 nm. 1 % (v/v) Triton X-100 was used to determine the maximal hemolysis and PBS was used as negative control.

### NO detection

Mouse peritoneal macrophages were incubated either with LPS (100 ng/ml, from *Escherichia coli* 0111:B4, Sigma-Aldrich, USA) and *Siba*Cec (0, 5, 10, and 20 μg/ml) dissolved in serum-free RPMI 1640 medium or incubated with *Siba*Cec (10 μg/ml) alone in 24-well plates (2.5 × 10^5^ cells/well) for 24 h. The cells incubated with serum-free RPMI 1640 and 100 ng/ml LPS were used as control. The culture medium was harvested to detect NO production. NO production was determined by detecting the nitrite level using Griess reagent (Beyotime, China) according to the manufacturer’s instructions.

### qPCR

Mouse peritoneal macrophages were cultured in 6-well plates (2 × 10^6^ cells/well) with RPMI 1640 (2 % FBS). The cells were incubated either with LPS (100 ng/ml) and *Siba*Cec (0, 5, 10, and 20 μg/ml) dissolved in serum-free RPMI 1640 medium or incubated with *Siba*Cec (10 μg/ml) alone. The cells incubated with serum-free RPMI 1640 and 100 ng/ml LPS were used as control. After treatment for 6 h, the cells were collected and total RNA was isolated. PrimeScript® Reverse Transcriptase (Takara, Japan) and SYBR green master mix (Takara, Japan) were used following the manufacturer’s instructions. qPCR was performed on a Realplex Mastercycler real-time PCR system (Eppendorf, Germany). The cycle counts of the target genes were normalized to the GAPDH gene, and accordingly the fold changes of the target genes were calculated. The primers used for qPCR are listed in Additional file 1: Table S1.

### Pro-inflammatory cytokine determination

Mouse peritoneal macrophages were incubated either with LPS (100 ng/ml) and *Siba*Cec (0, 5, 10, and 20 μg/ml) dissolved in serum-free RPMI 1640 medium or incubated with *Siba*Cec (10 μg/ml) alone in 24-well plates (2.5 × 10^5^ cells/well). The cells incubated with serum-free RPMI 1640 and 100 ng/ml LPS were used as control. After treatment for 6 h, the cell culture supernatants were collected and assessed for TNF-α, IL-1β, and IL-6 by using ELISA kits (Dakewei, China).

### Western blot analysis

Mouse peritoneal macrophages were incubated either with LPS (100 ng/ml) and *Siba*Cec (0, 5, 10, and 20 μg/ml) dissolved in serum-free RPMI 1640 medium or incubated with *Siba*Cec (10 μg/ml) alone in 6-well plates (2 × 10^6^ cells/well). The cells incubated with serum-free RPMI 1640 and 100 ng/ml LPS were used as control. After incubation for 30 min, the cells were washed twice with ice-cold PBS and lysed with RIPA lysis buffer (Beyotime, China). Then the cytoplasmic or nuclear proteins were extracted for Western blot analysis according to our previously described method [36]. Primary antibodies of phospho-ERK/ERK, phospho-p38/p38, NF-κB p65 (1:2000, Cell Signaling Technology, USA), GAPDH/Lamin B (1:5000, Santa Cruz Biotechnology, USA), and secondary antibody (1:5000, Cell Signaling Technology, USA) were used in Western blot analysis.

### Circular dichroism spectroscopy

CD spectroscopy was performed using a Jasco J-715 spectrophotometer (Jasco, Japan) to evaluate the secondary structure of *Siba*Cec in solvent environment. *Siba*Cec was dissolved in sodium dodecyl sulfate (SDS)/H_2_O solutions (0, 5, 10, 20, 40 mM) or LPS/H_2_O solution (0, 50, 100, 200, 400 ng/ml) to an ultimate concentration of 0.2 mg/ml. The spectra were measured at 298 K between 190 and 250 nm using 0.1 cm path-length cell with 1 nm bandwidth, 1 s response time, and a scan speed of 100 nm/min. Three consecutive scans were performed and averaged, followed by subtraction of the solvent signal.

### Dissociation of FITC-LPS aggregates

The ability of *Siba*Cec to dissociate LPS micelles was studied using FITC-conjugated LPS (1 μg/ml, Sigma-Aldrich, USA). FITC-LPS was excited at 480 nm and the change in the emission of FITC at 515 nm was monitored with different concentrations of *Siba*Cec (0, 50, 100, 200 μg/ml) dissolved in PBS. PBS was used as control. The fluorescence experiment was performed using a Cary Eclipse fluorescence spectrophotometer (Varian, Inc., USA).

### LPS neutralization assay

The ability of *Siba*Cec to neutralize LPS was assayed with the chromogenic LAL test which was performed according to the manufacturer’s instruction (GenScript, Nanjing, China). Briefly, different concentrations of *Siba*Cec (0, 25, 50, 100 μg/ml) dissolved in PBS were incubated with LPS at 37 °C for 30 min. PBS was used as control. After incubation, 100 μl of LAL solution was added to LPS-peptide mixtures (100 μl) in a pyrogen-free tube and incubated for 10 min followed by addition of pre-warmed substrate. After incubation of 6 min for the reaction, the absorbance was recorded at 545 nm on a microplate reader. The percentage of LPS neutralization was calculated as (*A*_blank_ − *A*_sample_)/*A*_blank_ × 100, where *A*_blank_ represents the absorbance of blank control (50 μl of LAL water + 50 μl of LPS solution).

### Bacterial feeding

Bacterial feed experiment was carried out as previously described [38]. In brief, the collected *S. bannaense* (~250 flies) were fed with 70 % sucrose solution *ad libitum.* After starving for 12 h, black flies were fed through cotton wool with 20 % sucrose solution (OD600 = 0.2) containing Gram-negative bacteria *E.coli* ATCC 25922. Total RNA was extracted from the salivary glands of immune stimulated or naive insects (sugar fed controls) at 12, 24, 36, 48 and 72 h after feeding. qPCR was performed to analyze the expression of *Siba*Cec mRNA as described above, with the housekeeping gene *β-actin* as an endogenous control.

### Statistics

Statistical analyses were performed using GraphPad Prism 5.0 (GraphPad Software Inc., San Diego, CA, USA) and Stata 10.0 software (StataCorporation, College Station, TX, USA). Data were presented as mean ± standard errors of mean, and compared using two-tailed equal variance Student’s *t*-test. *P < 0.05 and **P < 0.01 were considered as statistical significance.

## Results

### Characterization of *Siba*Cec

As illustrated in Fig. 1a, the fractions with antimicrobial activity (marked by A1) were collected, lyophilized, and further purified by C_18_ RP-HPLC. One peptide was purified from this step (Fig. 1b), and it was designated *Siba*Cec. After Edman degradation, the initial 22 N-terminal amino acid residues of *Siba*Cec was identified with the following sequence: GKLTKDKLKRGAKKALNVASKV. Due to the majority of insect cecropin-like peptides containing a C-terminally amidated residue, we presumed that the same structure feature exists in the *Siba*Cec. After carboxypeptidase Y treatment, no free amino acid can be detected by HPLC (data not shown). The result indicated that the C-terminal end of *Siba*Cec was amidated, which was further confirmed by mass spectrometry analysis. MALDI-TOF MS analysis (Fig. 1c) indicated that *Siba*Cec with the C-terminal amidation had a measured molecular mass of 3431.21 Da, matching well with the calculated molecular mass 3432.16 Da.

**Fig. 2:**
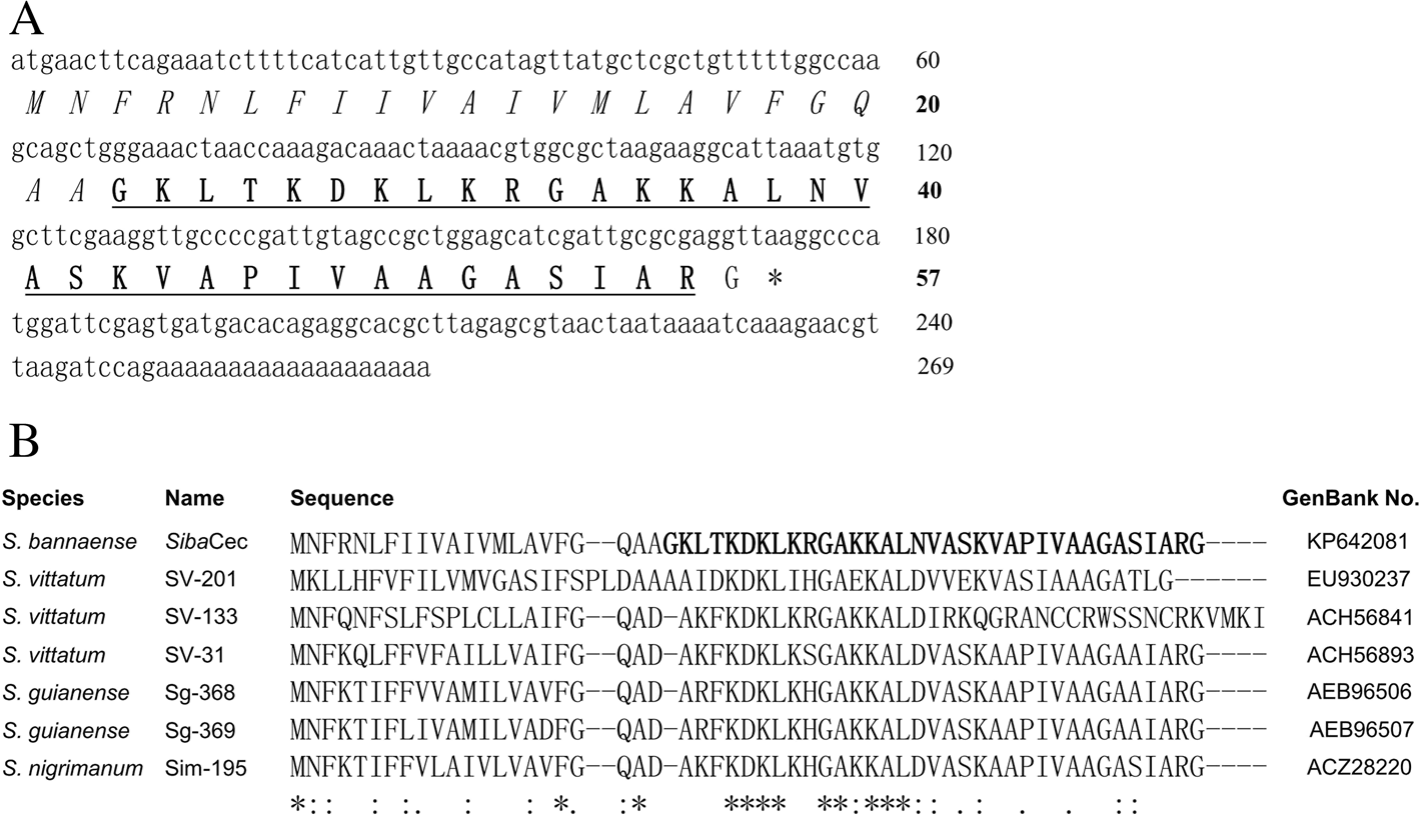
The cDNA sequence of *Siba*Cec precursor and the alignment of the amino acid sequence of *Siba*Cec with six known cecropin-like peptide precursors from three black fly species. **a** Deduced amino acid sequence is shown below the cDNA sequence. The putative signal peptide is italicized and the amino acid sequence of mature peptide is underlined and bold. The stop codon is indicated by an asterisk. Amino acid numbers or nucleotide numbers are shown after the sequences. **b** Six known cecropin-like peptide precursors in three black fly species are from Refs. [6–8]. The symbols under the alignment indicate: (*) identical sites; (:) conserved sites; (.) less conserved sites. Dashes are inserted to optimize the alignment

The cDNA clone encoding the precursor of *Siba*Cec was screened and sequenced from the salivary glands cDNA library of *S. bannaense* (GenBank accession number: KP642081). As shown in Fig. 2a, the deduced amino acid sequence of *Siba*Cec precursor is completely consistent with the result of Edman degradation sequencing. It is composed of 57 amino acid residues, including a predicted 22 amino acid signal peptide, a 34 amino acid mature peptide, and a C-terminal glycine for enzymic amidation. Analysis using the ExPASy MW/pI tool showed that *Siba*Cec has a predicted pI of 11.30. In addition, *Siba*Cec has the net charge of +8. BLAST search indicated that the *Siba*Cec precursor shared the highest identity of 70 % (40/57) with the salivary expressed cecropin precursor (GenBank accession number: ACH56893) from the black flies *S. vittatum*.

Multi-sequence alignment of the known cecropin-like peptide precursors from four black fly species (Fig. 2b) indicated that the putative signal peptide of these sequences are divergent, and nine amino acids residues are highly conserved within the mature peptide (Lys28, Asp29, Lys30, Leu31, Gla34, Ala35, Lys37, Ala38 and Leu39).

### Phylogenetic analysis of *Siba*Cec

**Fig. 3:**
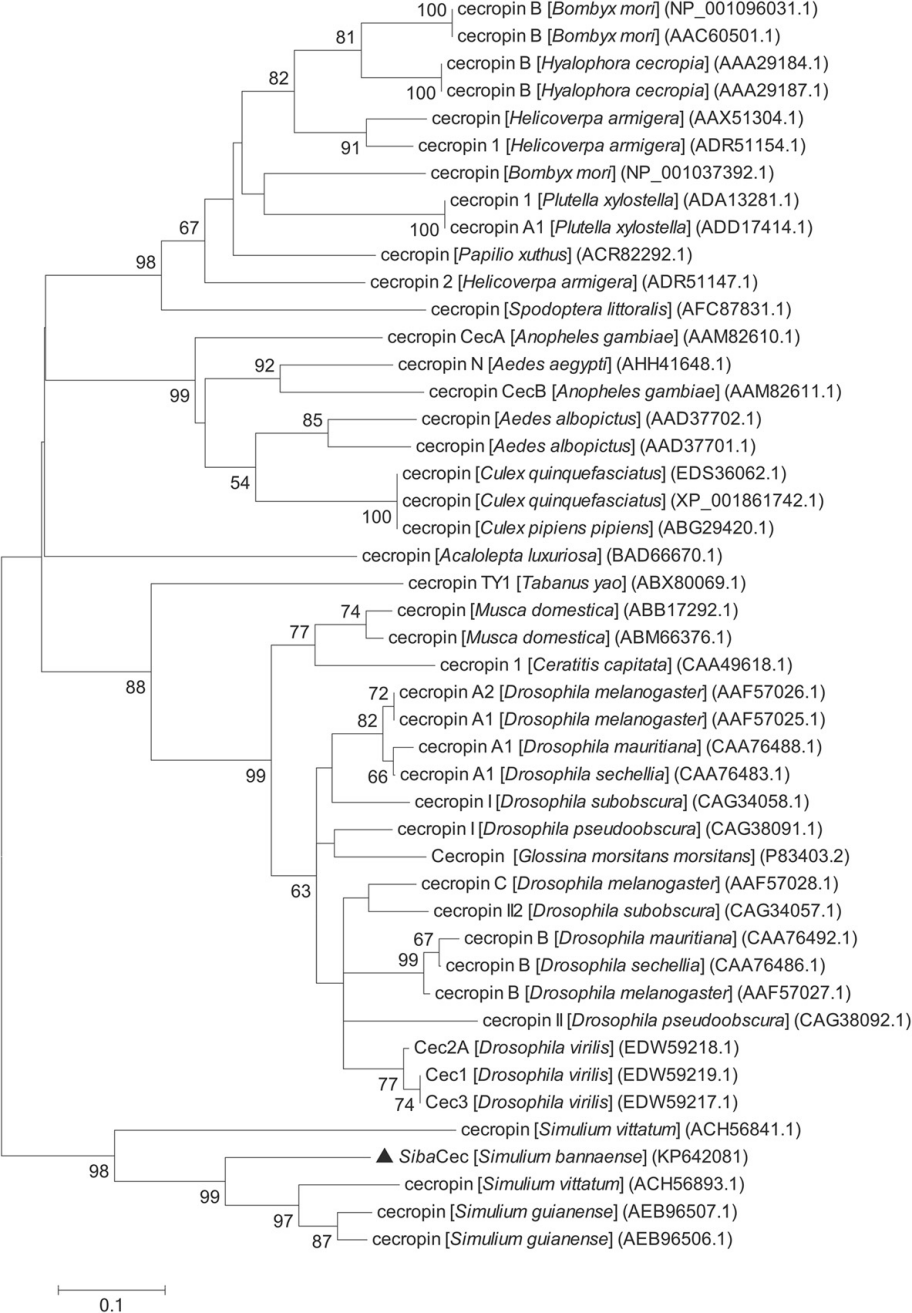
Phylogenetic tree based on the amino acid sequence of insect cecropins. The numbers on the branches represent the percent bootstrap support and only values over 50 % are shown. The bar at the bottom represents 5 % amino acid divergence. *Siba*Cec is indicated by a triangle

The phylogenetic tree was generated from the amino acid sequences of 46 insect cecropin precursors (28 insect species including 20 Diptera, 7 Lepidoptera, 1 Coleoptera). As showed in Fig. 3, the cecropin sequences are divided between two distinct groups: one cluster comprising 41 sequences derived from different insect orders (Diptera, Lepidoptera and Coleoptera) and the second cluster comprising the five sequences derived from Simuliidae, including *Siba*Cec.

### Antimicrobial activity of *Siba*Cec

The MICs of *Siba*Cec against Gram-positive and Gram-negative bacteria were determined. As listed in Table 1, *Siba*Cec exhibited broad spectrum antimicrobial activities against all ten bacterial strains tested, especially against Gram-negative bacteria *E.coli* (either standard strains or clinically isolated drug-resistance strains). *Siba*Cec showed much higher antimicrobial activities against Gram-negative bacteria (MICs ranging from 0.87 to 2.33 μM) than against Gram-positive bacteria (MICs ranging from 14.56 to 43.70 μM).

**Table 1:** Antimicrobial activity of *Siba*Cec

Microorganisms	MIC (μM)^a^
Gram-negative bacteria	
*Escherichia coli* ATCC 25922	0.87
*E. coli* clinical strain 1	1.45
*E. coli* clinical strain 2	1.45
*E. coli* clinical strain 3	2.33
*Pseudomonas aeruginosa* ATCC 9027	1.74
*Salmonella typhimurium* ATCC 14028	2.33
*Acinetobacter baumannii* ATCC 17978	2.33
Gram-positive bacteria	
*Staphylococcus aureus* ATCC 6538	43.70
*Bacillus subtilis* ATCC 6633	29.13
*Micrococcus luteus* ATCC 4698	14.56

^a^ *MIC* minimal inhibitory concentration. These MICs represent mean values of three independent experiments performed in duplicates

### *Siba*Cec alters the morphology of *E. coli*

**Fig. 4:**
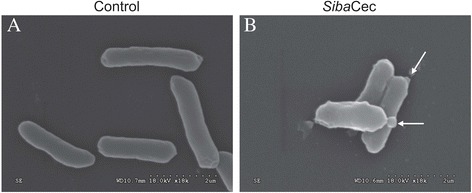
Scanning electron microscopy of bacteria treated with *Siba*Cec. **a** Control, *E.coli*. cells treated with PBS. **b** *E.coli* cells treated with *Siba*Cec (1 × MIC, 0.87 μM) dissolved in PBS. White arrow indicates damage to the plasma membranes of bacteria or the intracellular inclusions efflux

SEM was performed to study the possible mechanisms of action of *Siba*Cec on Gram-negative bacteria *E. coli* ATCC 25922. The cells treated with *Siba*Cec (1 × MIC) showed obvious morphological alterations by SEM analysis (Fig. 4). The membrane integrity of cells seemed to be disrupted, and cell shrinkage was obvious.

### *Siba*Cec shows low cytotoxicity and hemolytic activity

*Siba*Cec exhibited very low cytotoxicity toward the mouse peritoneal macrophages. At concentrations up to 200 μg/ml (58.27 μM), which is almost 67-fold higher than the MIC value of *Siba*Cec against *E.coli* ATCC 25922, *Siba*Cec induced cell death percentages as low as 4.26 %. As to hemolytic activity, *Siba*Cec yielded a hemolysis of 3.25 % at the same concentration of 200 μg/ml.

### *Siba*Cec reduces LPS-induced iNOS transcription and NO production

**Fig. 5:**
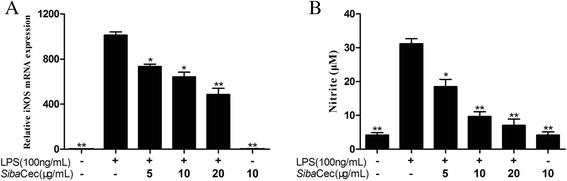
Effects of *Siba*Cec on iNOS transcription and NO production induced by LPS. **a** iNOS mRNA. **b** Nitrite production. Data are mean ± S.E. values from three independent experiments. *p <0.05, **p <0.01, significantly different compared with the control that treated with serum-free RPMI 1640 and 100 ng/ml LPS

To determine the effect of *Siba*Cec on the LPS-induced NO production in mouse peritoneal macrophages, we used qPCR to determine the mRNA level of iNOS. As shown in Fig. 5a, incubation with *Siba*Cec for 6 h significantly reduced the mRNA level of iNOS induced by 100 ng/ml LPS in a dose-dependent manner. At a concentration of 20 μg/ml, *Siba*Cec inhibited 51.9 % of the iNOS transcription. Furthermore, we determined the NO production by examining the nitrite concentration in the culture supernatants of mouse peritoneal macrophages. As illustrated in Fig. 5b, 100 ng/ml LPS induced 31.2 μM nitrate production. The addition of *Siba*Cec significantly reduced LPS-induced nitrite production. At the concentration of 20 μg/ml, *Siba*Cec inhibited 77.4 % of nitrite production

### *Siba*Cec inhibits LPS-induced pro-inflammatory cytokine production

**Fig. 6:**
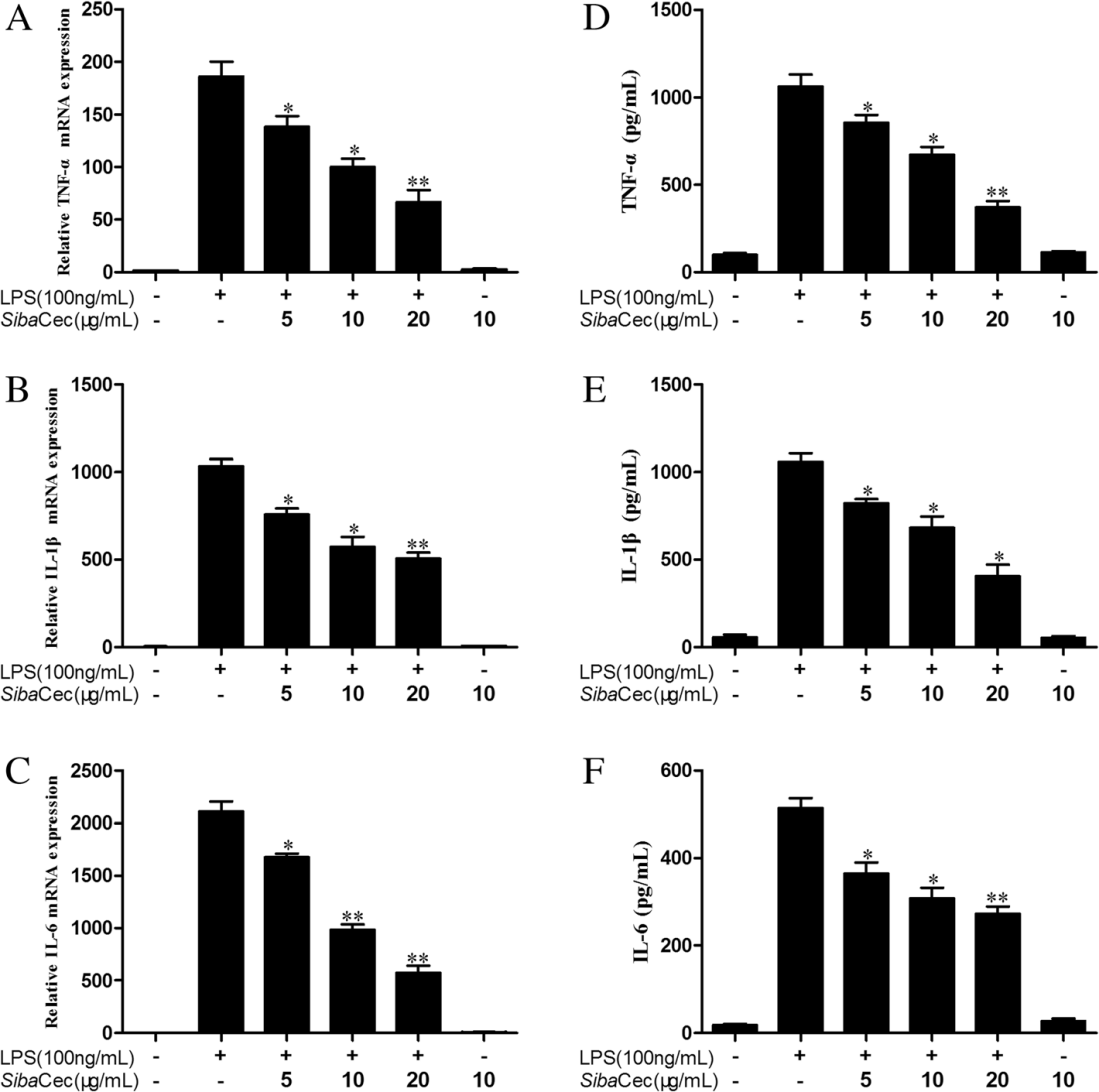
Effects of *Siba*Cec on pro-inflammatory cytokines transcription and secretion induced by LPS. **a** TNF-α mRNA. **b** TNF-α secretion. **c** IL-1β mRNA. **d** IL-1β secretion. **e** IL-6 mRNA. **f** IL-6 secretion. Data are mean ± S.E. values from three separate experiments. *p <0.05, **p <0.01, significantly different compared with the control that incubated with serum-free RPMI 1640 and 100 ng/ml LPS

To evaluate the effect of *Siba*Cec on LPS-induced pro-inflammatory cytokine production in mouse peritoneal macrophages, we used qPCR to determine pro-inflammatory cytokine gene expression. *Siba*Cec significantly blocked LPS-induced expression of TNF-α, IL-1β, and IL-6 in a dose-dependent manner. 20 μg/ml *Siba*Cec inhibited the expression of all three of the pro-inflammatory cytokine genes by 64.5, 51.4 and 68.8 %, respectively (Fig. 6a, b, and c). Furthermore, we used ELISA to confirm the effect of *Siba*Cec on pro-inflammatory cytokine production induced by LPS in mouse peritoneal macrophages. 100 ng/ml LPS alone induced the production of TNF-α, IL-1β, and IL-6 for about 1500, 1270, and 464 pg/ml, respectively. Fig. 6d, e and f showed a dose-dependent effect, which revealed activities similar to those obtained in the qPCR experiments. 20 μg/ml *Siba*Cec inhibited LPS-induced TNF-α, IL-1β, and IL-6 production by 65, 61.9, and 50.5 %, respectively.

### *Siba*Cec inhibits LPS-induced inflammatory response pathways

**Fig. 7:**
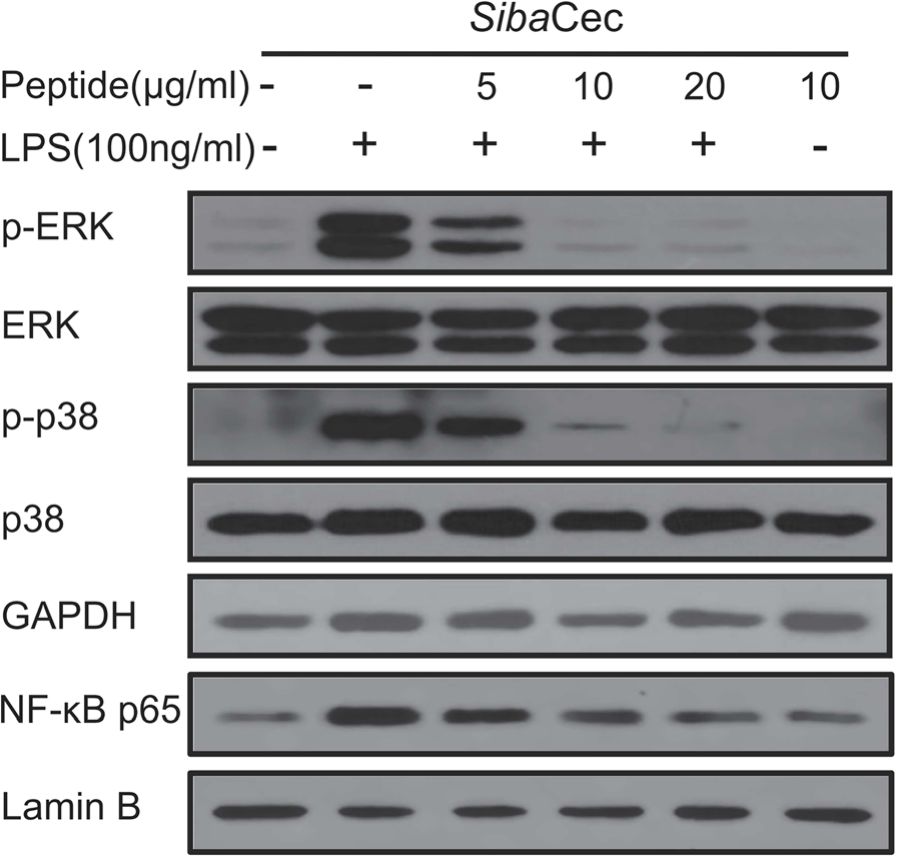
Effects of *Siba*Cec on LPS-induced inflammatory response pathways. Western blot of phosphorylation of ERK and p38, and the translocation of the NF-κB p65 subunit from cytoplasm to nucleus in peritoneal macrophages. The cells were incubated with LPS (100 ng/ml) and different concentrations of *Siba*Cec (5, 10 and 20 μg/ml). After incubation for 30 min, the cells were collected, and the cytoplasmic or nuclear proteins were extracted for Western blot analysis

The above mentioned data indicate that *Siba*Cec significantly inhibited the transcription and production of NO, TNF-α, IL-1β, and IL-6, which were induced by LPS in mouse peritoneal macrophages. MAPKs and NF-κB signal pathways play important roles in cytokine production. Therefore, we studied the effect of *Siba*Cec on LPS-induced inflammatory signaling pathways. As shown in Fig. 7, 100 ng/ml LPS induced the phosphorylation of ERK, p38, and the translocation of the NF-κB p65 subunit from cytoplasm to nucleus. The incubation of *Siba*Cec (10 μg/ml) markedly inhibited the LPS-induced phosphorylation of ERK and p38 and translocation of the NF-κB p65 subunit, which indicates that *Siba*Cec exerts its anti-inflammatory effect through inhibition of MAPKs and NF-κB inflammatory signaling pathways.

### Secondary structure of *Siba*Cec

**Fig. 8:**
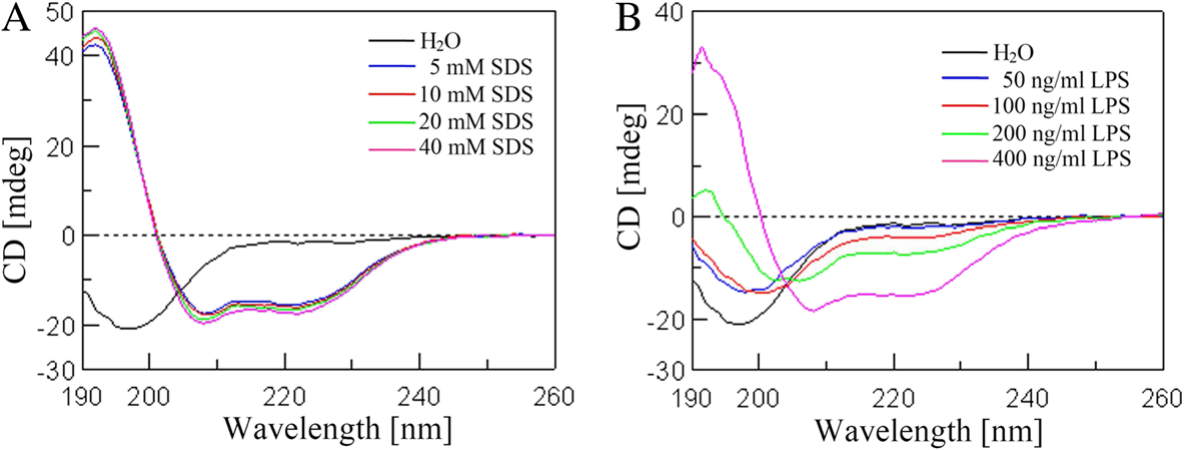
The CD spectra of *Siba*Cec in different solutions. **a** SDS/H2O solution (5, 10, 20, 40 mM). **b** LPS/H2O solution (50, 100, 200, 400 ng/ml). *Siba*Cec was dissolved in different solutions to an ultimate concentration of 0.2 mg/ml

To detect the secondary structures of *Siba*Cec in membrane-mimetic or hydrophobic environments, we analyzed the CD spectra of the peptide dissolved under increasing concentrations of SDS or LPS. As shown in Fig. 8, the CD spectra of *Siba*Cec dissolved in H_2_O showed a strong negative peak at 198 nm, which indicates that *Siba*Cec adopts a random coil conformation. In different concentrations of SDS solutions (5, 10, 40 mM), the CD spectra of *Siba*Cec exhibited a strong positive peak at 192 nm and two negative peaks at 208 and 222 nm (Fig. 8a), which indicates that *Siba*Cec mainly adopts an α-helix conformation in membrane-like environments.

In various proportions of LPS solutions (50, 100, 200, 400 ng/ml) (Fig. 8b), the conformation of *Siba*Cec gradually changed from a random-coil (percent decrease from 57.6 to 31.9) to an α-helix structure (percent increase from 0 to 35.1) in a relatively hydrophobic environment (Table 2).

**Table 2:** Secondary structural components of *Siba*Cec in different solutions

Solution	Helix (%)^a^	Beta (%)^a^	Turn (%)^a^	Random (%)^a^
H_2_O	0.0	15.3	25.3	59.4
SDS (mM)				
5	71.6	0.0	0.0	28.4
10	71.6	0.0	0.0	28.4
20	69.9	0.0	0.0	30.1
40	64.1	6.1	0.0	29.8
LPS (ng/ml)				
50	0.0	23.6	18.8	57.6
100	0.0	41.3	9.8	48.9
200	9.8	42.0	5.6	42.8
400	35.1	30.9	2.1	31.9

^a^Jasco-810 software was used to deconvolute CD spectra into fractional contents and these data are the average value of three scans

### *Siba*Cec dissociates FITC-LPS aggregates

**Fig. 9:**
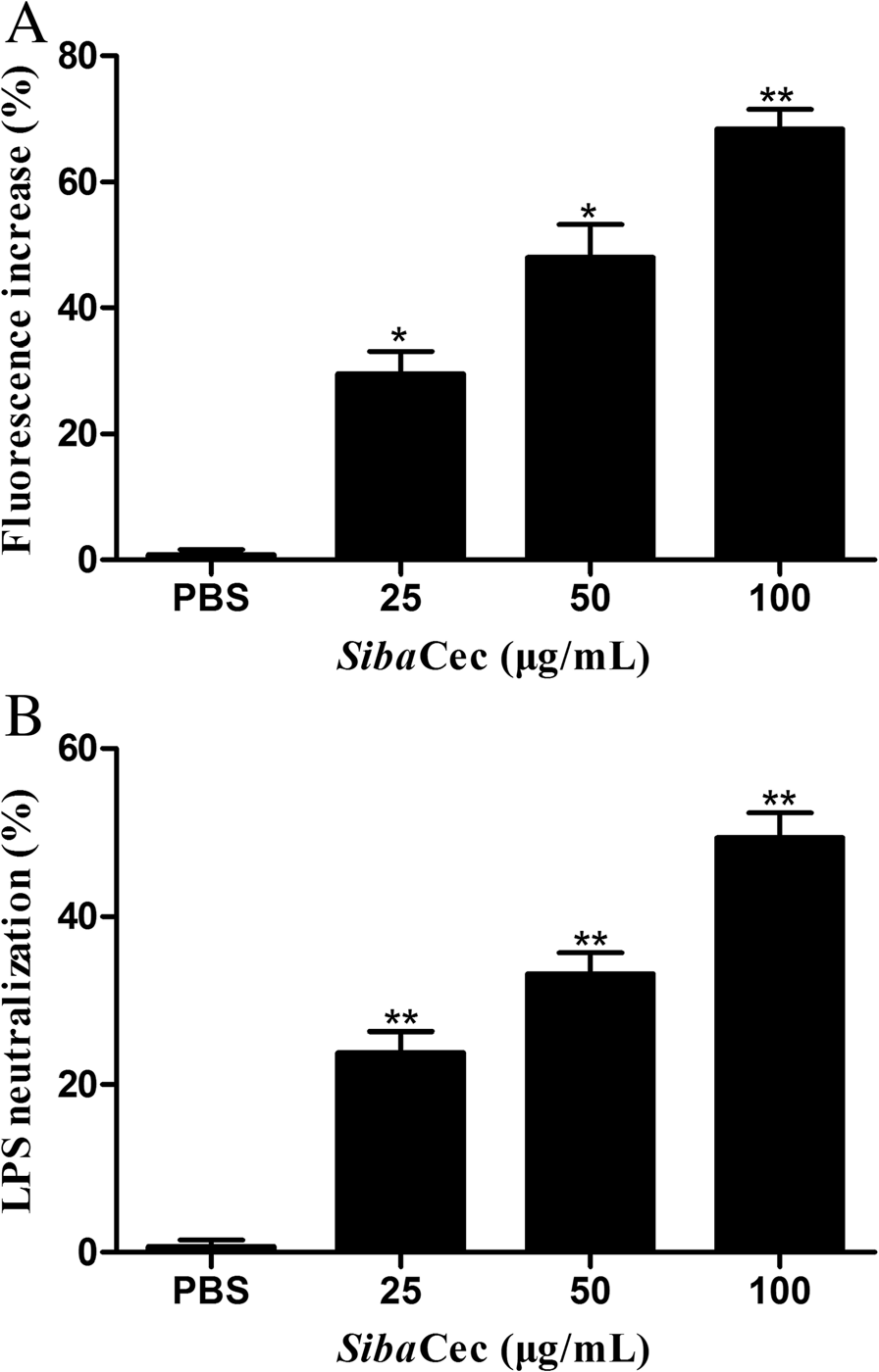
Interaction and neutralization of LPS by *Siba*Cec. **a** The changes in fluorescence intensity of FITC-labeled LPS as a function of various concentrations of *Siba*Cec, (**b**) LPS neutralization by *Siba*Cec. A chromogenic LAL assay was used to evaluate the neutralizing activity. Data are mean ± S.E. values from three separate experiments. *p <0.05, **p <0.01, significantly different compared with the control (PBS)

LPS forms micellar aggregates in water and FITC fluorescence is highly quenched in FITC-LPS micelles [29, 39]. Interactions of some AMPs with LPS may cause an enhancement of the FITC fluorescence because of de-quenching, which indicates the dissociation of large LPS aggregates into smaller sizes [39]. As shown in Fig. 9a, *Siba*Cec had a strong effect on the FITC-LPS aggregates, and the addition of *Siba*Cec caused a dose-dependent increase of FITC-LPS fluorescence. At the concentrations of 25, 50, 100 μg/ml, *Siba*Cec increased 29.6, 48.1 and 68.4 % of fluorescence intensity, respectively.

### *Siba*Cec neutralizes LPS

The chromogenic LAL assay was used to determine whether *Siba*Cec is active in neutralization of endotoxin. In parallel with the dissociation of FITC-LPS aggregates assay, *Siba*Cec exhibited LPS neutralizing activity in a concentration dependent manner (Fig. 9b). At the concentrations of 25, 50, 100 μg/ml, *Siba*Cec inhibited 23.8, 33.2 and 49.4 % of LPS, respectively.

### Transcript levels of *Sib*aCec increase after infection with *E.coli*

**Fig. 10:**
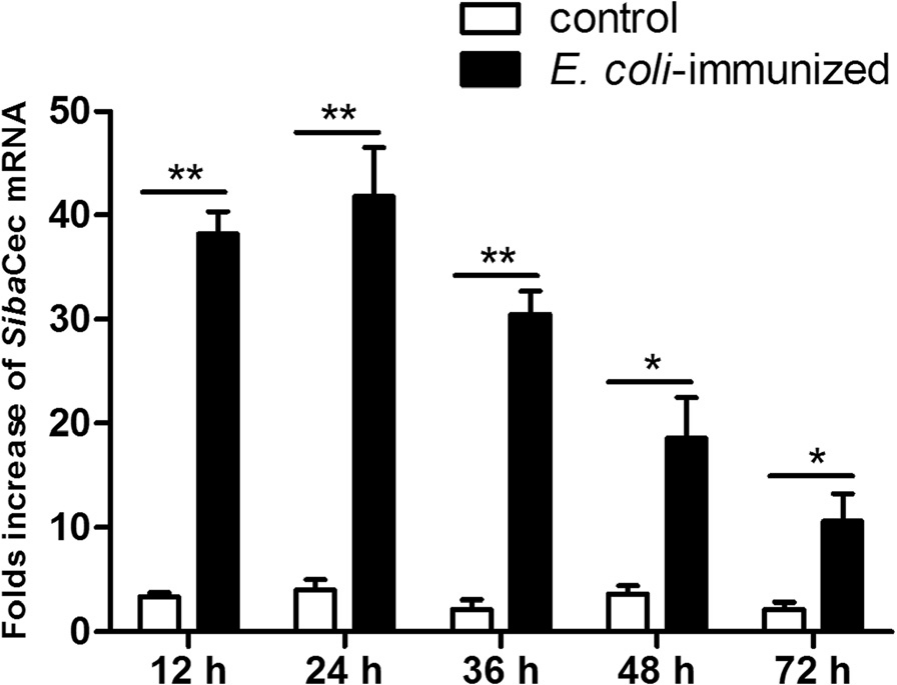
Fold increase of *Siba*Cec in the salivary glands of insects after oral infection with *E.coli* at different time course. Expression levels in the salivary glands of bacteria-immunized insects were calculated relative to the level of *Siba*Cec in corresponding naive insects, which was arbitrarily defined as 1. *p < 0.05, **p < 0.01, significantly different compared to the control that received the sucrose solution without *E.coli*

After *E. coli* ingestion, the expression levels of *Siba*Cec mRNA in the salivary glands of bacteria-immunized or naive insects were compared at the different time course, respectively. As illustrated in Fig. 10, the levels of *Siba*Cec mRNA were up-regulated by bacterial-challenge at 12, 24, 36, 48 and 72 h after *E. coli* ingestion (38.2, 41.8, 33.5, 29.6 and 15.6 fold, respectively). The expression of *Siba*Cec peaked at 24 h post-feeding and relatively decreased with time.

## Discussion

Black flies are blood-sucking insects that can secrete various immunomodulatory molecules to suppress the host’s inflammatory and immunologic reactions, and to contribute to efficient transmission of fly-borne pathogens [7]. The salivary gland extract of black fly *S. vittatum*, has been shown to contain immunomodulatory activities that reduces expression of I-A (mouse MHC class II), IL-5 and IL-10 in splenocytes [40, 41], and inhibits mitogen-stimulated mouse splenocyte proliferation [42]. However, the component(s) responsible for immunomodulation has not been characterized from black flies so far.

The current work identified a novel cationic cecropin-like peptide (*Siba*Cec) from the salivary glands of the black fly *S. bannaense*. The structural organization of *Siba*Cec precursor (Fig. 2a) is similar to other insect cecropin precursors, comprising a putative signal peptide and the mature peptide. The alanine residue (Ala22) in the precursor is cleaved to release the mature peptide, which is consistent with most cecropins isolated from Diptera [43]. *Siba*Cec presents a canonical feature of most characterized insect cecropins (an amidated C-terminus), but it is devoid of another feature (a tryptophan residue at position 1 or 2) [14]. The first two amino acid residues at the N-terminus of *Siba*Cec are glycine and lysine. The absence of tryptophan residue within the N-terminal domain was also reported in some mosquito cecropins [17, 43].

The phylogenetic tree of cecropin precursors (Fig. 3) showed that the black fly cecropins appear in one branch and other insect cecropins are grouped in another branch. This suggests the cecropins occurred in the insects before the divergence of the Diptera, Lepidoptera and Coleoptera. The result also supports that the cecropin molecules have evolved independently between these insect taxa [44].

*Siba*Cec, like other cationic AMPs, is a highly basic peptide with the net charge of +8, which implies that it would be readily attracted and adhered to the negative-charged bacterial surface. As expected, *Siba*Cec has a broad spectrum of antibacterial activity (Table 1), and it is more effective against Gram-negative bacteria (MICs ranging from 0.87 to 2.33 μM) than against Gram-positive bacteria (MICs ranging from 14.56 to 43.7 μM). SEM analysis indicated that such activities are carried out with a lytic effect on the bacterial membranes (Fig. 4). Additionally, CD spectroscopy indicated that *Siba*Cec mainly adopt an α-helical conformation in membrane-mimetic solutions (Fig. 8), which contributes to the ability of cationic peptide to kill bacteria. These results confirm that the microbial membrane is a key target for *Siba*Cec.

Extensive research has established that MAPK signal transduction pathways are involved in regulating the transcriptions of cytokine genes [29, 37]. In this study, *Siba*Cec significantly inhibited the transcription and production of pro-inflammatory factors induced by LPS in mouse peritoneal macrophages, including NO, TNF-α, IL-1β, and IL-6 (Figs. 5 and 6). Furthermore, Western blot analysis showed that *Siba*Cec significantly suppressed the LPS induced activation of ERK, p38, and the translocation of NF-κB p65 subunit (Fig. 7). These data indicated that *Siba*Cec executes the anti-inflammatory effect in LPS-stimulated murine macrophages by blocking the activation of MAPKs and NF-κB signaling pathways.

The analysis of interaction between *Siba*Cec and LPS indicated that *Siba*Cec can dissociate the aggregated form of LPS in a dose-dependent manner (Fig. 9a), and the LAL assay indicated that *Siba*Cec also can neutralize LPS in a dose-dependent fashion (Fig. 9b). Since *Siba*Cec is a membrane-targeting AMP with polycationic (basic-amino-acid-rich) and amphipathic α-helix structure, we rationally conclude *Siba*Cec binds to negatively charged LPS mostly through electrostatic interaction and the amphiphilic helical structure [45]. All these results implied that the neutralization of LPS by *Siba*Cec prevents LPS from binding to the LPS-binding protein, and hence suppresses the production of cytokines induced by LPS. Gram-negative bacteria and/or their endotoxins (LPS) may trigger a systemic inflammatory response, leading to some life-threatening systemic diseases, such as sepsis and septic shock [46]. The above results indicate that *Siba*Cec is not only involved in suppressing Gram-negative bacteria growth but also that it attenuates inflammatory responses induced by LPS. These functions of *Siba*Cec may favor resolution of infection and reverse potentially harmful inflammation.

The transcript levels of *Sib*aCec in the salivary glands of insects increased after oral infection with Gram-negative bacteria *E.coli*, reaching maximum at 24 h and then slowly decreased from that time point (Fig. 10). These data suggested that *Sib*aCec is involved in the innate humoral response of the black fly *S. bannaense.*

## Conclusions

In conclusion, the black fly cecropin-like peptide (*Siba*Cec) was identified in the present work by peptide purification and molecular cloning procedures. *Siba*Cec possesses potent antimicrobial activity against Gram-negative bacteria, and shows low cytotoxicity toward mammalian cells. It can neutralize LPS and exhibit strong anti-inflammatory activity. All the amalgamated properties make *Siba*Cec a potentially potent candidate for the treatment of inflammatory and infectious diseases.

## Additional file

Additional file 1: Table S1. Primer sequences used for cloning and qPCR in this study. (DOC 39 kb)

## References

[1] Adler PH, McCreadie JW, Mullen GR, Durden LA (2009). Black flies (Simuliidae). Medical and Veterinary Entomology.

[2] Ma D, Wang Y, Yang H, Wu J, An S, Gao L (2009). Anti-thrombosis repertoire of blood-feeding horsefly salivary glands. Mol Cell Proteomics.

[3] Xu X, Yang H, Ma D, Wu J, Wang Y, Song Y (2008). Toward an understanding of the molecular mechanism for successful blood feeding by coupling proteomics analysis with pharmacological testing of horsefly salivary glands. Mol Cell Proteomics.

[4] Calvo E, Mans BJ, Andersen JF, Ribeiro JM (2006). Function and evolution of a mosquito salivary protein family. J Biol Chem.

[5] Francischetti IM, Sa-Nunes A, Mans BJ, Santos IM, Ribeiro JM (2009). The role of saliva in tick feeding. Front Biosci (Landmark Ed).

[6] Chagas AC, Calvo E, Pimenta PF, Ribeiro JM (2011). An insight into the sialome of *Simulium guianense* (Diptera: Simuliidae), the main vector of river blindness disease in Brazil. BMC Genomics.

[7] Andersen JF, Pham VM, Meng Z, Champagne DE, Ribeiro JM (2009). Insight into the Sialome of the Black Fly, *Simulium vittatum*. J Proteome Res.

[8] Ribeiro JM, Valenzuela JG, Pham VM, Kleeman L, Barbian KD, Favreau AJ (2010). An insight into the Sialotranscriptome of *Simulium nigrimanum*, a black fly associated with Fogo Selvagem in South America. Am J Trop Med Hyg.

[9] Kläger SL, Watson A, Achukwi D, Hultmark D, Hagen HE (2002). Humoral immune response of *Simulium damnosum* s.l. following filarial and bacterial infections. Parasitology.

[10] Ham PJ, Albuquerque C, Baxter AJ, Chalk R, Hagen HE (1994). Approaches to vector control: new and trusted. 1. Humoral immune responses in blackfly and mosquito vectors of filariae. Trans R Soc Trop Med Hyg.

[11] Wei L, Mu L, Wang Y, Bian H, Li J, Lu Y (2015). Purification and characterization of a novel defensin from the salivary glands of the black fly, *Simulium bannaense*. Parasit Vectors.

[12] Koczulla AR, Bals R (2003). Antimicrobial peptides: current status and therapeutic potential. Drugs.

[13] Steiner H, Hultmark D, Engstrom A, Bennich H, Boman HG (1981). Sequence and specificity of two antibacterial proteins involved in insect immunity. Nature.

[14] Yi HY, Chowdhury M, Huang YD, Yu XQ (2014). Insect antimicrobial peptides and their applications. Appl Microbiol Biotechnol.

[15] Bulet P, Stöcklin R (2005). Insect antimicrobial peptides: structures, properties and gene regulation. Protein Pept Lett.

[16] Boman HG (2000). Innate immunity and the normal microflora. Immunol Rev.

[17] Vizioli J, Bulet P, Charlet M, Lowenberger C, Blass C, Muller HM (2000). Cloning and analysis of a cecropin gene from the malaria vector mosquito, *Anopheles gambiae*. Insect Mol Biol.

[18] Andra J, Berninghausen O, Leippe M (2001). Cecropins, antibacterial peptides from insects and mammals, are potently fungicidal against *Candida albicans*. Med Microbiol Immunol.

[19] Kokoza V, Ahmed A, Woon Shin S, Okafor N, Zou Z, Raikhel AS (2010). Blocking of plasmodium transmission by cooperative action of cecropin A and defensin A in transgenic *Aedes aegypti* mosquitoes. Proc Natl Acad Sci U S A.

[20] Luplertlop N, Surasombatpattana P, Patramool S, Dumas E, Wasinpiyamongkol L, Saune L (2011). Induction of a peptide with activity against a broad spectrum of pathogens in the *Aedes aegypti* salivary gland, following infection with Dengue Virus. PLoS Pathog.

[21] Niyonsaba F, Ushio H, Nakano N, Ng W, Sayama K, Hashimoto K (2007). Antimicrobial peptides human beta-defensins stimulate epidermal keratinocyte migration, proliferation and production of proinflammatory cytokines and chemokines. J Invest Dermatol.

[22] Heilborn JD, Nilsson MF, Kratz G, Weber G, Sorensen O, Borregaard N (2003). The cathelicidin anti-microbial peptide LL-37 is involved in re-epithelialization of human skin wounds and is lacking in chronic ulcer epithelium. J Invest Dermatol.

[23] Nizet V, Ohtake T, Lauth X, Trowbridge J, Rudisill J, Dorschner RA (2001). Innate antimicrobial peptide protects the skin from invasive bacterial infection. Nature.

[24] Lai Y, Gallo RL (2009). AMPed up immunity: how antimicrobial peptides have multiple roles in immune defense. Trends Immunol.

[25] Bowdish DM, Davidson DJ, Scott MG, Hancock RE (2005). Immunomodulatory activities of small host defense peptides. Antimicrob Agents Chemother.

[26] Vieira CS, Waniek PJ, Mattos DP, Castro DP, Mello CB, Ratcliffe NA (2014). Humoral responses in *Rhodnius prolixus*: bacterial feeding induces differential patterns of antibacterial activity and enhances mRNA levels of antimicrobial peptides in the midgut. Parasit Vectors.

[27] Vieira CS, Mattos DP, Waniek PJ, Santangelo JM, Figueiredo MB, Gumiel M (2015). *Rhodnius prolixus* interaction with Trypanosoma rangeli: modulation of the immune system and microbiota population. Parasit Vectors.

[28] Nayduch D, Lee MB, Saski CA (2014). Gene discovery and differential expression analysis of humoral immune response elements in female *Culicoides sonorensis* (Diptera: Ceratopogonidae). Parasit Vectors.

[29] Kim JK, Lee E, Shin S, Jeong KW, Lee JY, Bae SY (2011). Structure and function of papiliocin with antimicrobial and anti-inflammatory activities isolated from the swallowtail butterfly, *Papilio xuthus*. J Biol Chem.

[30] Lee E, Shin A, Kim Y (2015). Anti-inflammatory activities of cecropin A and its mechanism of action. Arch Insect Biochem Physiol.

[31] Scott MG, Rosenberger CM, Gold MR, Finlay BB, Hancock RE (2000). An alpha- helical cationic antimicrobial peptide selectively modulates macrophage responses to lipopolysaccharide and directly alters macrophage gene expression. J Immunol.

[32] Ryu S, Choi SY, Acharya S, Chun YJ, Gurley C, Park Y (2011). Antimicrobial and anti-inflammatory effects of Cecropin A(1–8)- Magainin2(1–12) hybrid peptide analog p5 against Malassezia furfur infection in human keratinocytes. J Invest Dermatol.

[33] Liu Y, Xia X, Xu L, Wang Y (2013). Design of hybrid β-hairpin peptides with enhanced cell specificity and potent anti-inflammatory activity. Biomaterials.

[34] Bjellqvist B, Hughes GJ, Pasquali C, Paquet N, Ravier F, Sancez JC (1993). The focusing positions of polypeptides in immobilized pH gradients can be predicted from their amino acid sequences. Electrophoresis.

[35] Chenna R, Sugawara H, Koike T, Lopez R, Gibson TJ, Higgins DG (2003). Multiple sequence alignment with the Clustal series of programs. Nucleic Acids Res.

[36] Wei L, Che H, Han Y, Lv J, Mu L, Lv L (2015). The first anionic defensin from amphibians. Amino Acids.

[37] Wu J, Wang Y, Liu H, Yang H, Ma D, Li J (2010). Two immune-regulatory peptides with antioxidant activity from tick salivary glands. J Biol Chem.

[38] Telleria EL, Sant’Anna MR, Alkurbi MO, Pitaluga AN, Dillon RJ, Traub-Csekö YM (2013). Bacterial feeding, Leishmania infection and distinct infection routes induce differential defensin expression in *Lutzomyia longipalpis*. Parasit Vectors.

[39] Rosenfeld Y, Papo N, Shai Y (2006). Endotoxin (lipopolysaccharide) neutralization by innate immunity host-defense peptides. Peptide properties and plausible modes of action. J Biol Chem.

[40] Cross ML, Cupp MS, Cupp EW, Galloway AL, Enriquez FJ (1993). Modulation of murine immunological responses by salivary gland extract of *Simulium vittatum* (Diptera: Simuliidae). J Med Entomol.

[41] Cross ML, Cupp EW, Enriquez FJ (1994). Modulation of murine cellular immune responses and cytokines by salivary gland extract of the black fly *Simulium vittatum*. Trop Med Parasitol.

[42] Tsujimoto H, Gray EW, Champagne DE (2012). Black fly salivary gland extract inhibits proliferation and induces apoptosis in murine splenocytes. Parasite Immunol.

[43] Lowenberger C, Charlet M, Vizioli J, Kamal S, Richman A, Christensen BM (1999). Antimicrobial activity spectrum, cDNA cloning, and mRNA expression of a newly isolated member of the cecropin family from the mosquito vector *Aedes aegypti*. J Biol Chem.

[44] Tassanakajon A, Somboonwiwat K, Amparyup P (2015). Sequence diversity and evolution of antimicrobial peptides in invertebrates. Dev Comp Immunol.

[45] Stewart I, Schluter PJ, Shaw GR (2006). Cyanobacterial lipopolysaccharides and human health - a review. Environ Health.

[46] Schulte W, Bernhagen J, Bucala R (2013). Cytokines in sepsis: potent immunoregulators and potential therapeutic targets-an updated view. Mediators Inflamm.

