# Supplementary material for: A cecropin-like antimicrobial peptide with anti-inflammatory activity from the black fly salivary glands
Source: Parasit Vectors. 2015 Oct 24;8:561. doi: 10.1186/s13071-015-1176-8 (PMC4620007; doi:10.1186/s13071-015-1176-8)
Supplement: Additional file 1: Table S1. — Primer sequences used for cloning and qPCR in this study. (DOC 39 kb) [file 13071_2015_1176_MOESM1_ESM.doc]

**Additional file1: Table S1.** Primer sequences used for cloning and qPCR in this study

| **Primer** | **Sequence (5’→3’)** | **application** |
| --- | --- | --- |
| *Siba*Cec-F_1_ | AARMGIGGIGCNAARAARGC* | 3’ end screening |
| 3’ PCR primer | CGGGGTACGATGAGACACCA | 3’ end screening |
| *Siba*Cec-R_1_ | GATGCTCCAGCGGCTACAAT | 5’ end screening |
| 5’ PCR primer | AAGCAGTGGTATCAACGCAGAGT | 5’ end screening |
| *Siba*Cec-F_2_ | CTGGGAAACTAACCAAAGACA | qPCR |
| *Siba*Cec-R_2_ | GCTCTAAGCGTGCCTCTGTGT | qPCR |
| *Actin*-F | TGTTGTCACTGTACGCCTCCG | qPCR |
| *Actin*-R | TGATGTCGCGAACGATTTCCC | qPCR |
| TNF-α-F | CGGTGCCTATGTCTCAGCCT | qPCR |
| TNF-α-R | GAGGGTCTGGGCCATAGAAC | qPCR |
| IL-1β-F | ATGGCAACTGTTCCTGAACTC | qPCR |
| IL-1β-R | GCCCATACTTTAGGAAGACA | qPCR |
| IL-6-F | AGTTGCCTTCTTGGGACTGA | qPCR |
| IL-6-R | TCCACGATTTCCCAGAGAAC | qPCR |
| iNOS-F | CTGCAGCACTTGGATCAGGAACCTG | qPCR |
| iNOS -R | GGAGTAGCCTGTGTGCACCTGGAA | qPCR |
| GAPDH-F | GTGAAGGTCGGTGTGAACGGATT | qPCR |
| GAPDH-R | GGAGATGATGACCCTTTTGGCTC | qPCR |

*****Where R stands for A or G, M stands for A or C, N stands for A, C, G or T, and I stands for hypoxanthine.
